# Bilateral subgaleal hematoma after a robot-assisted radical prostatectomy: an uncommon complication

**DOI:** 10.23938/ASSN.1047

**Published:** 2023-08-31

**Authors:** Iñigo Rubio Baines, Antonio Martínez Simón, Francisco Javier Ancizu, Isidro Olavide, Cristina Honorato-Cía

**Affiliations:** 1 Universidad de Navarra Clinica Universidad de Navarra Pamplona Spain; 2 Universidad de Navarra Clinica Universidad de Navarra Pamplona Spain

**Keywords:** Robot-assisted radical prostatectomy, Subgaleal hematoma, Steep Trendelenburg, Prostatectomía robótica radical, Hematoma subgaleal, Trendelenburg extremo

## Abstract

Robot-assisted radical prostatectomy is a relatively recent technique. Its advantages include less invasiveness and better pain management, but has specific anesthesia requirements, such as steep Trendelenburg position and pneumoperitoneum. Mild complications are common, e.g., transient hypotension or soft tissue edema.

We present a case of a 62-year old male who developed subgaleal hematoma associated with transient neurologic impairment after surgery. Jugular vein insufficiency was suspected as the most likely cause. The patient recovered fully.

Robot-assisted radical prostatectomy can be a challenging procedure due to the anesthesia requirements, but most complications are mild and transient. However, patients should be carefully assessed before surgery. We identified potential factors that may have led to this complication: the abnormal prolonged surgical time, the steep Trendelenburg, a non-assessed jugular vein insufficiency, and/or patient’s obesity.

## RESUMEN

La prostatectomía robótica radical es una técnica relativamente reciente. Sus ventajas son una menor invasividad y mejor control del dolor. Tiene algunas peculiaridades anestésicas, como Trendelenburg extremo y neumoperitoneo. Son frecuentes las complicaciones leves, como la hipotensión transitoria o el edema de partes blandas.

Presentamos el caso de un varón de 62 años que desarrolló un hematoma subgaleal asociado a deterioro neurológico transitorio tras la intervención. Se sospechó como causa más probable una incompetencia venosa yugular. El paciente se recuperó completamente.

La prostatectomía robótica radical puede ser un reto debido a los requisitos anestésicos, pero la mayoría de las complicaciones son leves y transitorias. No obstante, los pacientes deben ser estudiados cuidadosamente antes de la intervención. En este caso podemos encontrar varios factores que pueden haber influido, como el tiempo quirúrgico anormalmente prolongado, el Trendelenburg extremo, una insuficiencia de la vena yugular no evaluada y/o la obesidad del paciente.

## INTRODUCTION

Robot-assisted radical prostatectomy is a relatively recent technique. Its advantages include less invasiveness, better pain management, and reduced length of hospital stay^1^. The surgical time, once the learning curve is over, is about two hours.

Some of the requirements when using this technique can be challenging for the anesthesiologist. The patient must be positioned in steep Trendelenburg (> 30º), a pneumoperitoneum established, and a deep muscular blockade is desirable. This potentially helps impair homeostasis, ventilation, and cervical and cephalic venous-lymphatic drainage. The presence of increased intraocular or intracranial pressures is a contraindication for performing this technique.

Common risks of the steep Trendelenburg position, which increase with time, include intraoperative hypotension, and soft-tissue edema (tongue, eyelids, conjunctiva, and scalp); severe complications are rare.

In this case report, we communicate an infrequent complication: subgaleal hematoma associated with neurologic impairment.

## CASE REPORT

Sixty-two-year old male with a history of high blood pressure, hypertensive cardiomyopathy, type 2 diabetes, dyslipidemia, and obesity (105 kg, body mass index 35 kg/m^2^). The patient had undergone a bladder electroporation in 2018, and had had symptoms of a potential salicylate sensitivity, which was not further evaluated.

Preoperative blood tests showed low platelet count (140x10^9^/L; normal range (NR): 150-450x10^9^/L), normal prothrombin time 12.2 s (NR: 11-15 s), low international normalized ratio (INR) (0.9; NR: 1-1.2), and activated partial thromboplastin clotting time (aPTT) 32.1 s and ratio 1.0 (NR: 0.9-1.2). The patient had no history of prolonged bleeding with minor injuries or frequent or disproportionate bruises. There was no family history of coagulopathy or coagulation factor deficiency.

The robot-assisted radical prostatectomy lasted much longer than usual (9 h 52 min - 532 min) due to technical difficulties linked to obesity, with no other incidents during the procedure. As expected, there was some degree of edema, and once the table was returned to the base position (0º) we observed that the tongue, eyelids, and conjunctiva were more edematous than usual. The leak test was negative and the patient was admitted to the intensive care unit overnight suspecting important upper airway edema.

**Figure 1:**
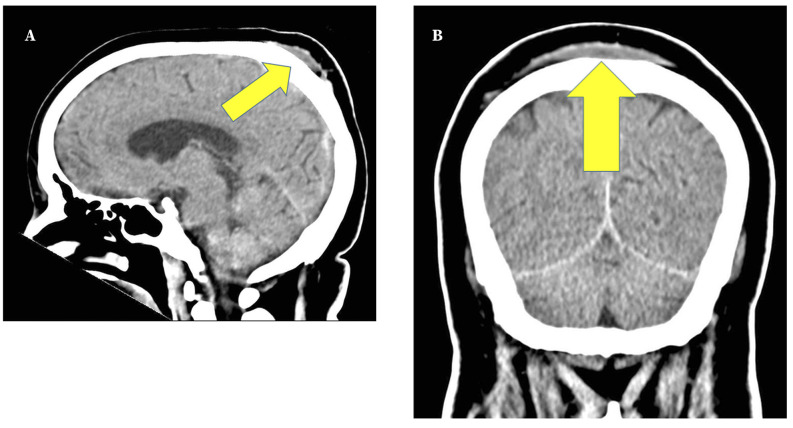
Computerized tomography scan of the brain. **A**. Sagittal view. Posterior subgaleal hematoma (yellow arrow). **B**. Coronal view. Bilateral parietal subgaleal hematoma (yellow arrow).

The patient was intubated overnight and received steroids for the edema (dexamethasone 4 mg/8 h). The following morning, he was weaned from the ventilator and extubated without problems. The neurologic examination showed a fully responsive, oriented person who followed commands and with no deficits in sensitivity or strength. About three hours after extubation, the patient became apneic and desaturated quickly to < 70% and stopped responding. Manual ventilation with a bag valve mask was delivered and oxygen saturation went back to normal within seconds; the patient quickly started responding and breathing spontaneously again. Immediate neurologic examination revealed the onset of lower left limb paresthesia and mild bilateral miosis. Blood gases showed respiratory acidosis (pH = 7.24), with increased partial arterial carbon dioxide pressure (PaCO_2_) (50 mm Hg) and increased partial arterial oxygen pressure (PaO_2_) (after bag valve mask ventilation).

An electroencephalogram and computerized tomography (CT) scan of the brain were requested; no abnormalities were found. As an incidental finding, the CT scan showed a significant bilateral parietal subgaleal hematoma (Fig. 1), initially deemed secondary to the intraoperative positioning, as there were no other plausible recent reasons that could explain it.

**Figure 2:**
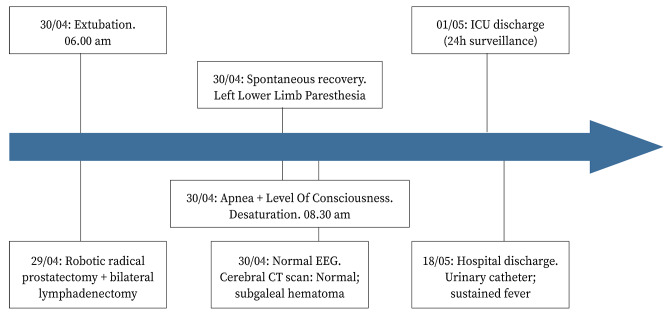
Case timeline.

The hematoma was treated conservatively and resorbed uneventfully after a few days. A control CT was considered unnecessary. The lower left limb paresthesia disappeared in minutes after regaining consciousness (Fig. 2).

We were unable to ascertain the cause of the event immediately. An unnoticed medication bolus was ruled out after careful checking. There might have been some degree of airway obstruction due to remaining edema. We also hypothesized central apnea secondary to increased intracranial pressure, assuming that internal jugular vein valve incompetence (IJVVI) could have contributed and played a role in the development of the subgaleal hematoma.

Patient is being followed by the urology department in our center, and two years after surgery he is living a normal life. By the time this manuscript was written, patient´s prostate-specific antigen (PSA) level was 0.028 ng/mL (NR: 0-4 ng/mL) and he did not present any neurological symptoms.

## DISCUSSION

A robot-assisted prostatectomy can be challenging for the anesthesiologist due to the combination of steep Trendelenburg (>30º) and pneumoperitoneum^2^, as they may affect ventilatory pressures and cervical-cephalic venous and lymphatic drainage (soft-tissue edema). Deep neuromuscular blockade is required to help ventilation and, because the robot works with mechanical arms fixed to the human body, any sudden or unexpected movement may cause serious injuries. It is important to consider the increased access difficulties associated to this procedure.

Previous conditions such as increased intracranial pressure or high intraocular pressure^3^, or other health problems like obesity, ischemic heart disease, or restrictive pulmonary diseases, may be considered relative contraindications, and risk-benefit should be carefully assessed in each case^4^.

According to the literature, in cases of IJVVI, considerable intracranial pressure increase may occur during the steep Trendelenburg position (optic nerve sheath diameter can increase more than 1.5 mm). Over the first postoperative days, agitation, disorientation, and lower scores in neurological scales, e.g., the Mini Mental State Examination, have been described^5^. While the episode of decreased level of consciousness seen in our patient may be related to IJVVI, its assessment is not part of the standard preoperative testing for this surgery, so we have no data to support this claim. An intraoperative echocardiographic IJVVI study should have been carried out.

Our next suspicion was acute ischemia. Cerebral blood flow does not seem to be affected by steep Trendelenburg^6^, so if this had been an ischemic episode - ≥ 12 h after surgery - it is probably unrelated to the position of the patient. The computed tomography scan rules out acute bleeding or ischemia, but shows a spontaneous bilateral parietal subgaleal hematoma, although it does not explain the loss of consciousness, but may have been caused by the surgical position.

Lymphatic and venous drainage are certainly affected by steep Trendelenburg, and soft-tissue edema (conjunctiva, eyelid, tongue, and even upper airway) is a common and well-known side effect of even slight head-down positions, especially if they are prolonged in time, as was our case. Usually, the edema resolves spontaneously within hours after correcting the position, and permanent deficits are infrequent. It is difficult to ascertain whether this played a role in the development of the hematoma. Most probably, the loss of consciousness and the hematoma are unrelated, and a combination of several factors, including remaining airway edema and residual sedation, may have caused the episode.

Cerebral edema has been reported elsewhere after robot-assisted hysterectomy; the patient was agitated and extubation was difficult. A CT scan on the second postoperative day showed brain swelling^7^. In our case, the CT scan did not show any degree of edema.

Robot-assisted radical prostatectomy presents very specific challenges to the anesthesiologist due to the combination of pneumoperitoneum and steep Trendelenburg (> 30º). This position, particularly for an extended period of time, may cause significant edema in the airway and soft tissues in the head and neck. Airflow may be compromised, visual problems due to conjunctival edema may develop, and there are reports of neurological impairment following this procedure. Abnormal prolonged surgical time, steep Trendelenburg, unassessed jugular vein insufficiency, and patient`s obesity must be taken into consideration as potential risk factors for bilateral subgaleal hematoma.
